# Analysis of 2-year spherical equivalent progression in emmetropic children with non-cycloplegic refraction: a retrospective chart review

**DOI:** 10.1186/s12886-023-02869-6

**Published:** 2023-03-30

**Authors:** Yoo Jin Kim, Tae Gi Kim

**Affiliations:** 1 Department of Ophthalmology, Graduate School, Kyung Hee University, Seoul, Korea; 2 Department of Ophthalmology, Kyung Hee University Hospital at Gangdong, Kyung Hee University, Seoul, Korea

**Keywords:** Myopia, Cycloplegic refraction, Emmetropia, Non-cycloplegic refraction, Spherical equivalent progression

## Abstract

**Background:**

We aimed to investigate children with an emmetropic non-cycloplegic refraction (NCR) to compare the difference in progression of NC spherical equivalent (SE) over 2 years between the children with emmetropic and hyperopic cycloplegic refraction (CR) values.

**Methods:**

Through a retrospective medical record review, 59 children aged under 10 years were evaluated. Refractive error was calculated as the average of the SE values of both eyes. According to the CR results, children with emmetropia (-0.50 to 1.00 diopter [D]) were assigned to group 1 (n = 29), and those with hyperopia (≥ 1.00 D) were assigned to group 2 (n = 30). The prevalence of myopia and SE progression were compared over 2 years. Correlations between final SE progression and baseline age and refractive error were analyzed and multiple regression analysis was conducted. Receiver operating characteristic curves that achieved the best cutoff points to distinguish between the groups were calculated.

**Results:**

Group 1 showed significantly myopic SE changes compared to baseline at the 1-year follow-up, and group 1 was significantly myopic compared with group 2 at the 2-year follow-up. Myopia prevalence was 51.7% in group 1 and 6.7% in group 2 after 1 year, and 61.1% and 16.7% after 2 years, respectively. In the correlation analysis, baseline age, baseline CR, and difference between CR and NCR showed significant correlations with the 2-year SE progression (r = -0.359, p = 0.005; r = 0.450, p < 0.001; r = -0.562, p < 0.001, respectively). However, NCR refractive error showed no significant correlation (r = -0.097, p = 0.468). In multiple regression analysis, baseline age (β= -0.082), and CR-NCR difference (β= -0.214) showed a significant effect on SE progression for 2 years. When an NCR value of 0.20 D was set as the cut-off value to distinguish between the groups, a sensitivity of 70% and specificity of 92% were obtained.

**Conclusion:**

Even if NCR showed emmetropia, children with baseline CR values of emmetropia showed greater SE progression compared with those with hyperopia. Cycloplegia is essential to confirm the correct refractive status in children. It may be useful for predicting prognosis of SE progression.

## Introduction

Myopia, or nearsightedness, is a common eye condition that causes blurred vision. This occurs because axial elongation of the eyeball makes the image focus in front of the retina [1]. The occurrence and progression of myopia has long been studied and, it is considered to be a multifactorial disorder affected by both genetic and environmental factors [2, 3]. As a factor related to the progression of myopia, excessive accommodation by a shorter reading distance is speculated to affect the progression of myopia, but the exact relationship between excessive accommodation and myopia progression is not clear [4–6].

Clinically, excessive accommodation can lead to an overestimation of myopia under non-cycloplegic refraction (NCR), which is due to a transient spasm of the ciliary muscle that alters the shape of the crystalline lens, causing an increase in the refractive power of the eye. This error can be reduced or eliminated with cycloplegia. Therefore, an evaluation of the refractive error under cycloplegia is considered to be the standard, especially in children [7]. Through examining the difference in refractive error before and after cycloplegia, the degree of a patient’s accommodative tone can be indirectly determined. There are studies which have reported that the difference between cycloplegic and non-cycloplegic autorefraction is associated with myopia progression. Lin et al. reported that greater refractive error difference before and after cycloplegia was associated with the progression of refractive error among myopic children, but not with the onset of myopia [8]. Thus, cycloplegic refraction (CR) is important for diagnosing refractive errors, correction, and vision improvement and for estimating SE progression.

However, although it is agreed that CR is essential in children, side effects caused by cycloplegia, such as difficulty in near work, photophobia, and long waiting times, may occur [9]. In actual clinical practice, when emmetropic refraction results are obtained from NCR, and uncorrected visual acuity is good, whether CR should be performed in these children is debatable.

This study aimed to assess the necessity for the CR in children with an emmetropic NCR by comparing the difference in SE progression between children with emmetropic CR and hyperopic CR over a 2-year period.

## Methods

### Study design and patients

This study was approved by the Institutional Review Board of Kyung Hee University Hospital at Gangdong (approval number: 2021-10-015). An exemption for the requirement for informed consent was granted by the Institutional Review Board of Kyung Hee University Hospital at Gangdong due to the retrospective design of the study. We reviewed the medical records of children under 10 years of age who visited the outpatient department and cooperated for autorefraction at the Kyung Hee University Hospital at Gangdong, Seoul, Korea, between January 2010 and December 2020. Among them, 59 children who showed emmetropia in NCR and were able to be followed up for 2 years were included. The exclusion criteria included wearing contact or ortho-K lenses, using eye drops for any type of ocular diseases and having an amblyopia or history of ocular surgery. According to CR, children with emmetropia were classified into group 1, while children with hyperopia were classified into group 2. SE was calculated as the spherical power of the refractive error plus one-half of the cylinder power. Hyperopia was defined as a spherical equivalent (SE) values of ≥ + 1.00 diopter (D) and emmetropia as a SE ranging between 0.99 and − 0.49 D. Myopia was defined as a SE refraction of ≤ -0.50 D. We analyzed the mean SE values of both eyes. Regarding the SE values used for comparative analysis, NCR was used for baseline and 1- and 2-year follow-up measures. CR was used only to differentiate between group 1 and group 2 at baseline. SE progression was defined as the change in refractive error in the direction of the negative diopter over time.

### Patient examinations

All children received a NCR and CR (Canon RK-F1, Canon Inc., Tokyo, Japan), with an average of three consecutive readings. For cycloplegia, 1% cyclopentolate (Alcon, Fort Worth, TX, USA) and 0.5% tropicamide/0.5% phenylephrine mixed eye drops (Mydrin-P, Santen, Osaka, Japan) were administered to each eye three times with a 5-min interval. After 30 min, eyes were checked for pupillary light reflection to determine whether cycloplegia is completed. If the pupil was dilated to 6 mm or more, the eye was considered as cycloplegic.

### Statistical analysis

Statistical analysis was performed using the independent samples t-test in SPSS version 18.0 software (SPSS Inc., Chicago, USA). To confirm the correlation between the refractive values of the right and left eye, a correlation analysis was performed and the difference between the left and right refractive values was analyzed. The difference between the left and right refractive values was analyzed and was not significant, thus the mean SE values of both eyes were used in the analysis. Comparisons between baseline non-cycloplegic SE and non-cycloplegic SE after 1 and 2 years were analyzed using one-way ANOVA test, and comparisons of the 2-year SE progression rate between the two groups were analyzed using an independent t-test. Correlation and multiple regression analyses were performed on factors that showed an association with SE progression over two years. Pearson correlation was used to analyze the correlation of SE changes with the baseline age, NCR value, CR value and the difference between CR and NCR. Multiple regression analysis was performed to determine whether baseline age, NCR, CR and CR-NCR differences could affect SE progression at 2 years. The analysis method was selected stepwise. Receiver operating characteristic curves that achieved the best cutoff points to distinguish between groups 1 and 2 were calculated. In order to confirm the cut off value that can distinguish group 1 and group 2 via the NCR results, binomial logistic regression was performed and subsequently area under the curve (AUC) values were calculated, and sensitivity and specificity were determined. A specificity of 90% was set to compare the sensitivities. A p-value of less than 0.05 was considered statistically significant.

## Results

### Baseline patients demographics

A total of 59 children (26 boys, 33 girls) were included in the study; 29 in group 1 and 30 in group 2. There were no statistically significant differences between the two groups in terms of age, uncorrected visual acuity, and best-corrected visual acuity (p > 0.05) Baseline demographics are shown in Table 1. Both groups showed significantly hyperopic changes in the CR compared to the NCR. After CR, a hyperopic shift of 0.55 ± 0.64 D in group 1 and 1.70 ± 0.92 D in group 2 was observed. Baseline CR value was significantly hyperopic in group 2 compared to group 1 (p < 0.001). NCR values of right and left eye were 0.16 ± 0.52 D, 0.14 ± 0.52 D, and CR values were 1.44 ± 1.23 D, 1.27 ± 1.13 D, respectively. There was a no statistically significant difference between the right and left eyes both before and after cycloplegia (*p* = 0.632; 95% CI -0.05 to 0.09, cohen’s d = 0.039, *p* = 0.197; 95% CI -0.09 to 0.43, cohen’s d = 0.144, respectively). There was a statistically significant correlation between the right and left eyes before and after cycloplegia. (r = 0.861, *p* < 0.001, r = 0.648, *p* < 0.001, respectively)

**Table 1:** Baseline characteristics of the children

	Group 1 Emmetropia with CR ( n = 29) (mean ± SD)	Group 2 Hyperopia with CR (n = 30) (mean ± SD)	P value	Effect size (Cohen’s = d)	95% CI
Sex (M : F)	13:16	14:16	NA	NA	NA
Age	6.28 ± 1.51	5.94 ± 1.15	0.519	0.25	-0.36 to 1.03
UCVA (logMAR)	0.17 ± 0.08	0.19 ± 0.07	0.283	-0.27	-0.06 to 0.12
BCVA (logMAR)	0.11 ± 0.07	0.12 ± 0.07	0.409	-0.14	-0.52 to 0.02
NCR (Diopter)	-0.17 ± 0.35	0.39 ± 0.47	0.000	-1.34	-0.77 to -0.34
CR (Diopter)	0.37 ± 0.43	2.09 ± 0.74	0.000	-2.83	-2.05 to -1.40
NCR-CR difference (Diopter)	-0.55 ± 0.64	-1.70 ± 0.92	0.000	1.45	0.81 to 1.53

NCR = Non-cycloplegic refraction; CR = Cycloplegic refraction

### Two year SE progression

**Fig. 1:**
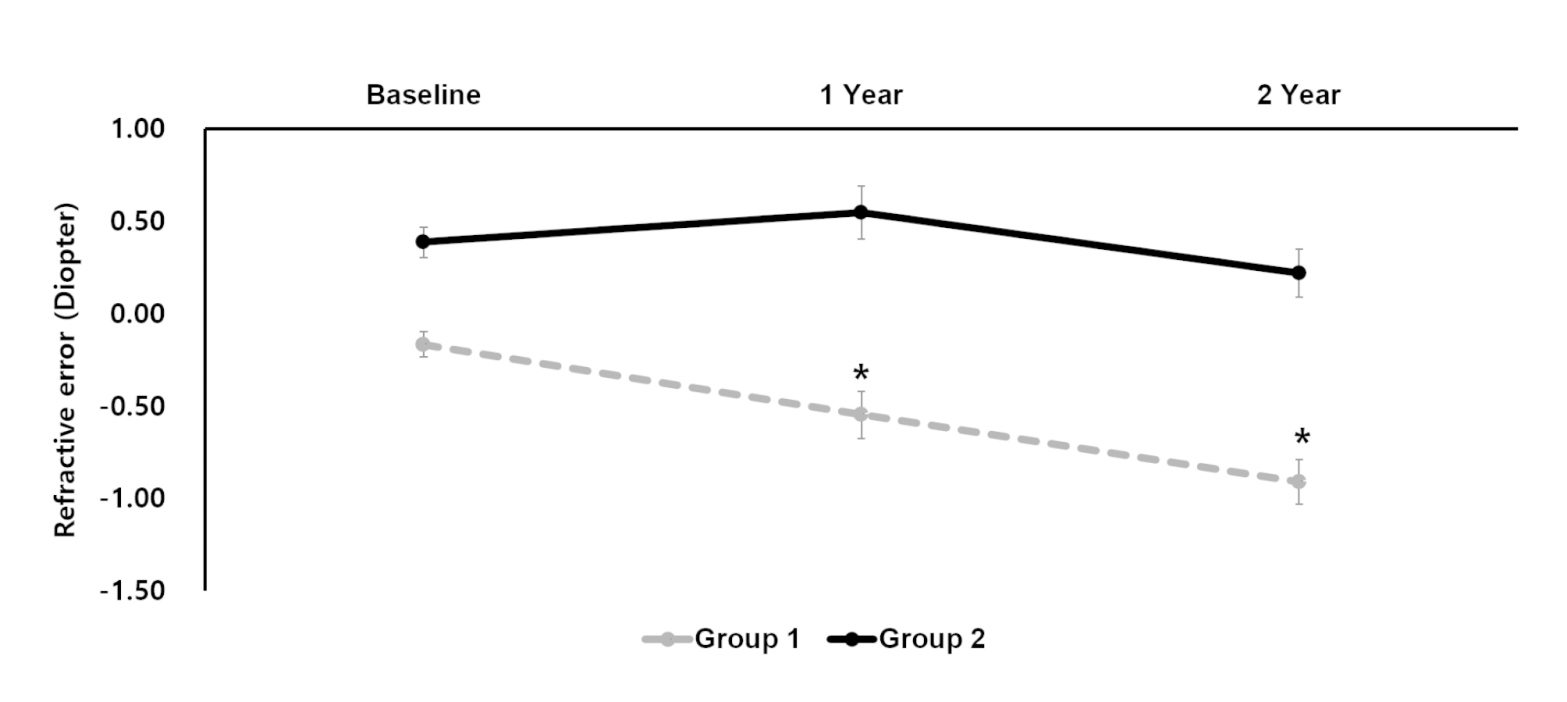
Mean non-cycloplegic spherical equivalent of the two groups during 2 years of follow-up. Group 1: Emmetropia after cycloplegic refraction; Group 2: Hyperopia after cycloplegic refraction. A single asterisk indicates a significant difference (p < 0.05) compared to baseline. *p value by Games-Howell (post-hoc) test

In one-way ANOVA, group 1 showed a statistically significant result with F(2, 84) = 11.844, *p* < 0.001, showing a difference in NCR values during the follow-up period compared with baseline NCR. Using the Games-Howell test, there was a statistically significant difference between baseline NCR values after 1 year and between baseline NCR and NCR values after 2 years (*p* = 0.032, *p* < 0.001, respectively) (Fig. 1). However, there was no statistical significance in group 2 with F(2, 87) = 1.864, *p* = 0.160. Therefore, there was no difference between NCR values during the follow-up period.

**Fig. 2:**
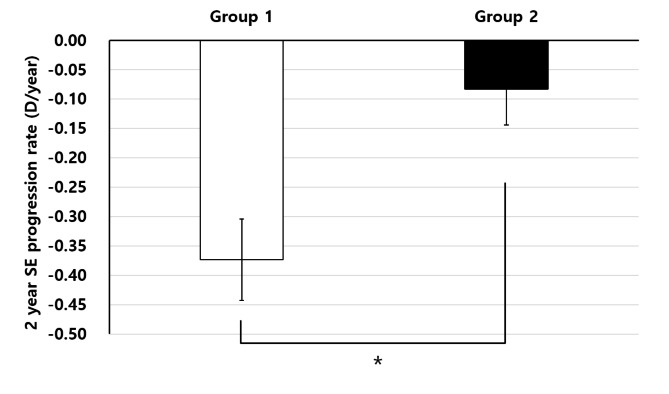
Rate of spherical equivalent (SE) progression of the two groups. Group 1: Emmetropia after cycloplegic refraction; Group 2: Hyperopia after cycloplegic refraction. P-values were calculated by independent t-tests. *P < 0.05

**Fig. 3:**
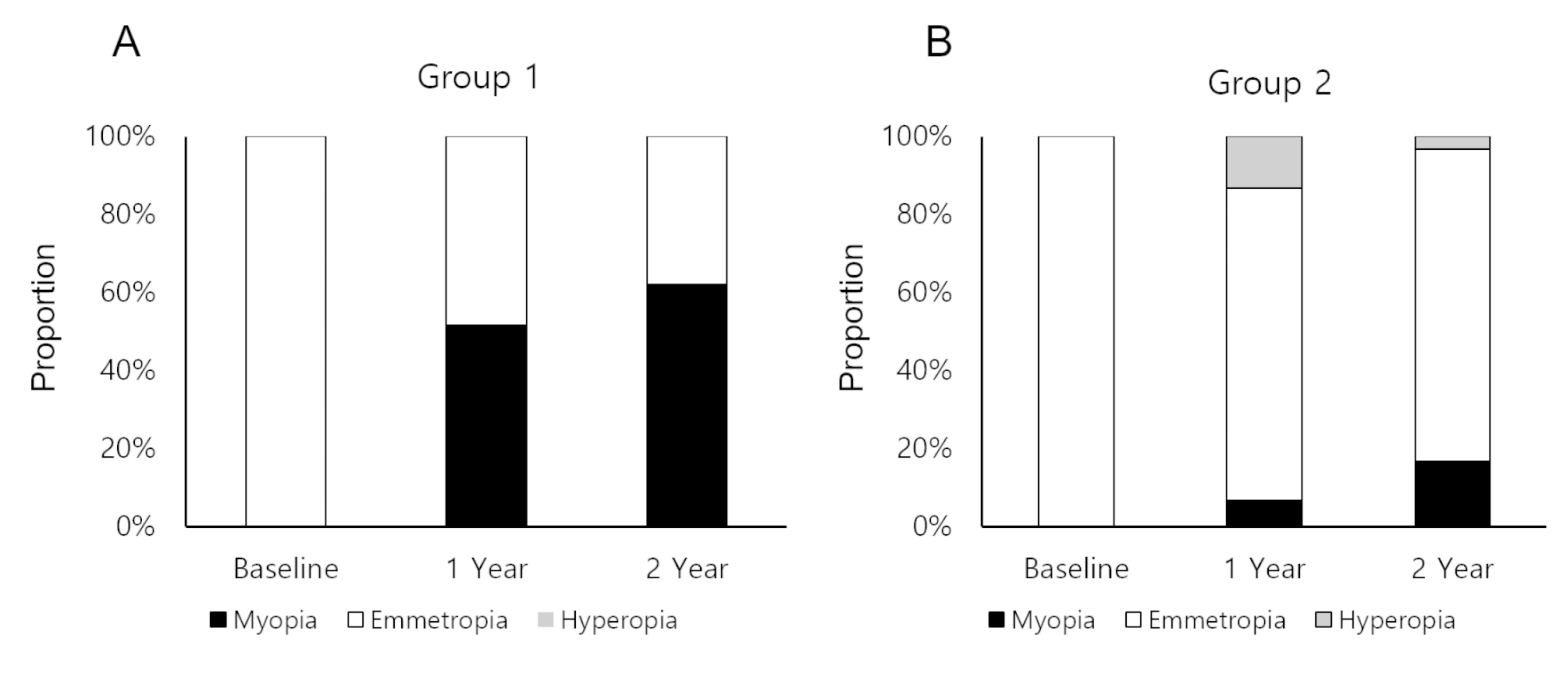
Prevalence of refractive error of the two groups during 2 years of follow-up. Group 1: Emmetropia after cycloplegic refraction; Group 2: Hyperopia after cycloplegic refraction. Myopia: ≤ SE -0.50 D; Emmetropia: SE between − 0.50 D and 1.00 D; Hyperopia: SE ≥ 1.00 D by non-cycloplegic refraction

The rate of SE progression for the 2 years of follow-up was − 0.37 ± 0.31 D/year in group 1 and − 0.08 ± 0.41 D/year in group 2, showing a statistically significant difference between the groups (p < 0.001) (Fig. 2). The annual prevalence of myopia in group 1 was 51.7% after 1 year and 61.1% after 2 years, whereas in group 2, myopia was observed in 6.7% and 16.7% of participants, respectively (Fig. 3).

### Pearson correlation and multiple linear regression

**Fig. 4:**
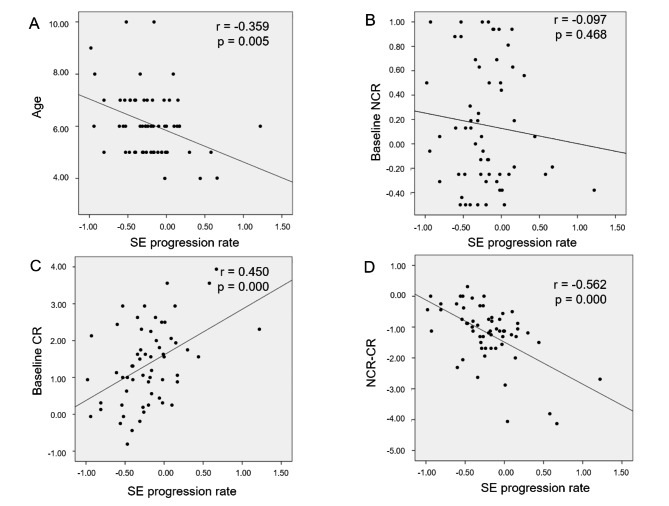
Correlation between baseline age (A), non-cycloplegic refraction (NCR) (B), cycloplegic refraction (CR) (C), NCR-CR difference, and the 2-year SE progression rate. P-values were calculated by Pearson’s correlation test

In the correlation analysis, baseline age, CR, and the difference between CR and NCR were correlated with SE progression over the 2 years. The older the baseline age, the more myopia via CR, and the smaller the difference between the CR and NCR, the greater the SE progression (Fig. 4). However, baseline NCR did not show a significant correlation with SE progression.

In multiple regression analysis, the result of F(2, 56) = 17.926, *p <* 0.001 showed that the regression model was significant, and adj.R2 = 0.369 showed 36.9% explanatory power. Baseline age showed β= -0.082 (*p* = 0.011), and CR-NCR difference showed β= -0.214 (*p* < 0.001), which had a significant effect on SE progression for 2 years. However, baseline NCR and CR showed β=-0.90 (p = 0.395) and β = 0.192 (p = 0.396), respectively, and did not significantly affect SE progression.

### Receiver operating characteristic curves

**Fig. 5:**
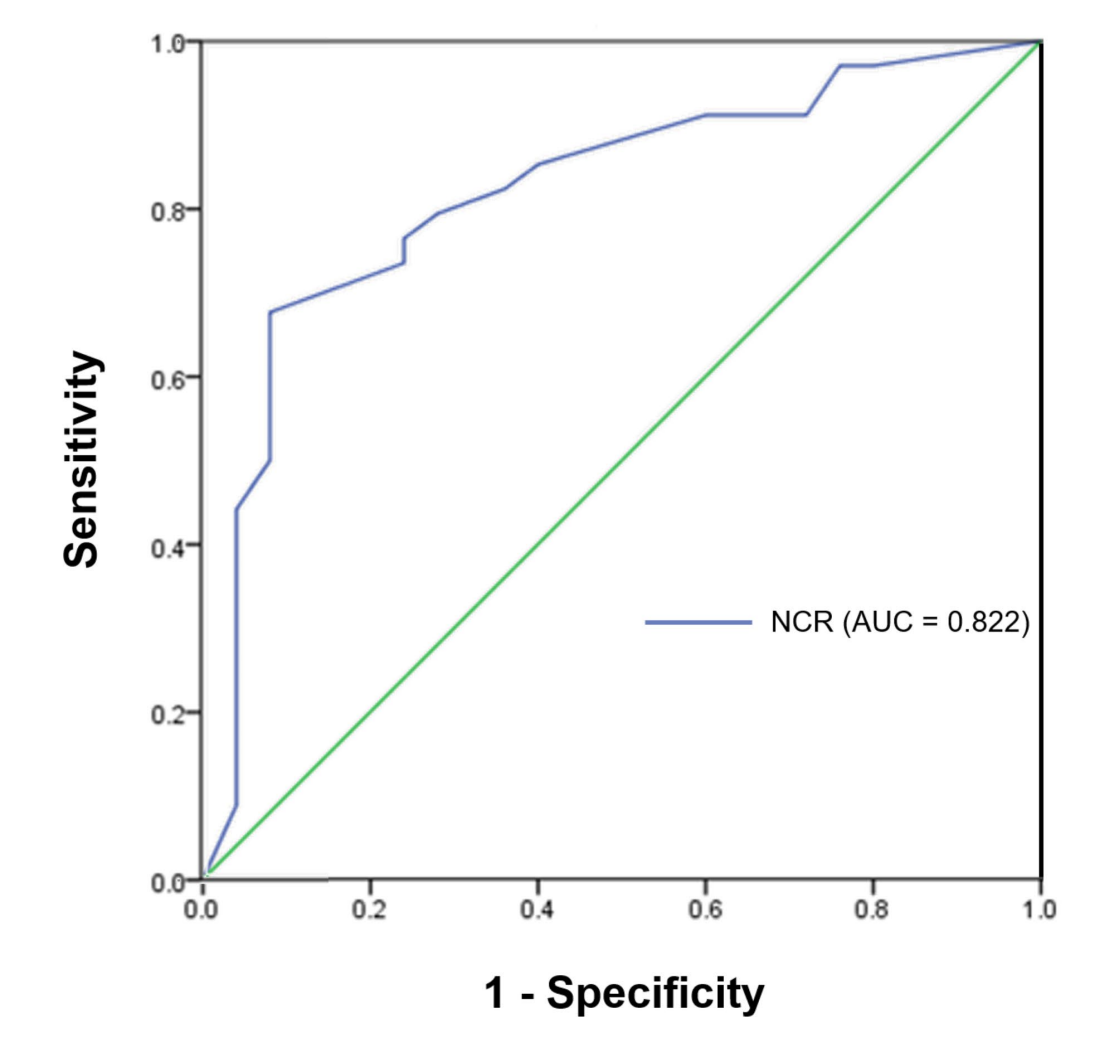
Receiver operating characteristic curve (ROC) for prediction of cycloplegic hyperopia based non-cycloplegic refraction (NCR).

In binomial logistic regression analysis, baseline NCR was found to be statistically significantly able to distinguish between group 2 and group 1 (odds ratio = 20.671, *p* < 0.001). The predictive performance of using NCR refractive error alone to distinguish group 1 and 2 show an AUC value of 0.822 (Fig. 5). When the cut-off value was set as 0.20 D, a sensitivity of 70% and a specificity of 92% were observed.

## Discussion

In general, NCR in children shows lower hyperopia or higher myopia than does CR because the ciliary muscle tone remains higher in children than in adults [10–12]. Therefore, CR is considered the gold standard for measuring true refractive errors, particularly in young children. It was reported that lack of cycloplegia would lead to significant misclassification of myopia, emmetropia and hyperopia even in young adult. Furthermore, the importance of cycloplegia was emphasized in epidemiologic studies [13]. However, after cycloplegia, decreased near vision loss and photophobia may occur, and the cycloplegic test takes more than 30 min. Therefore, if visual acuity is good and emmetropia is seen via NCR, it is often difficult to decide whether to perform CR. In this study, the prognosis of myopia according to CR results was analyzed to confirm the necessity of CR for children who show emmetropia in NCR. As in previous studies, NCR showed more myopic refraction values than CR, and the difference between NCR and CR was statistically significant in both groups 1 and 2. Zhao et al. [10] reported an average difference of 1.23 D between CR and NCR in the total refractive error type, and Fotouhi et al. [11] reported a 0.71 D hyperopic shift in 5–10-year-old children, which are similar to the results of our study.

In this study, the SE progression was significantly greater in group 1 than in group 2 at the 1- and 2-year follow-up. In correlation analysis also showed that when the CR value was less hyperopic and the difference between the NCR and CR was small, the progression of SE was greater. This means that SE progression was less when the baseline CR value was hyperopic. Although multiple linear regression showed that the baseline CR value did not affect the 2-year SE progression, the NCR-CR difference was found to significantly affect the 2-year SE progression. This result also implies the importance of CR, and the more hyperopic the CR value compared to NCR, the more delayed the 2 year SE progression.

The prevalence of myopia also showed a similar trend. After 2 years of follow-up, 61.1% of group 1 showed myopia, whereas 16.7% of group 2 had myopia. Lin et al. reported that in myopic children, the greater the CR-NCR difference, the greater the progression of myopia [8]. This is the opposite of our result. However, in our study, we enrolled emmetropia and hyperopic patients with cycloplegia, not myopia, and it is presumed that Lin et al.‘s study showed different results from our study for this reason. Near work is associated with SE progression due to an overactive or excessive accommodative response that primarily occurs after sustained near work [14–20]. In this study, it was assumed that the difference between CR and NCR reflects the degree of accommodation, but it may have shown different results because this may not be completely consistent with the accommodation that actually occurs during near work. On the other hand, some study has reported that near work-induced transient myopia was significantly associated with the progression of myopic refractive shift in hyperopia but not in myopia [21].

SE progression has a multifactorial process that involves factors other than accommodation. According to previous reports, myopia has been found to be related to very complex processes such as accommodation as well as subsequent choroid changes or retinal blur from the lag of accommodation and color cues to accommodation [22]. Therefore, additional research is needed on the difference between CR and NCR, and the degree of accommodation acting in actual near work. In this study, the greater the CR-NCR difference, the less SE progression occurs. From our results, we speculate that the baseline CR value had an effect on the final SE progression rather than the accommodation effect. Although there is no previous study with the same design as the current study, Hirsch reported that even if the manifest retinoscopy value at 5–6 years of age was not myopia, it is shown that the more the refraction value was hyperopic, the less myopia occurred [23]. He also reported that in cases with 0.50 to + 1.25 D at 5–6 years of age, they are most likely to be emmetropic at the age of 13–14 years. Therefore, confirming the current refractive error can be helpful in predicting the occurrence of myopia in the future, and CR is necessary to accurately diagnose the current refractive error.

The correlation and multiple regression analysis showed that the older the baseline age, the more SE progressed. As the eyeball grows with age, the axial length increases, and myopia progresses in proportion to this. It has known that juvenile-onset myopia typically develops between 6 and 12 years of age [24–28]. Although eyeball size increases in all children, it is not yet fully understood why the axial length increases beyond emmetropia only in myopia. Considering that myopia is caused by an increase in axial length, in children with short axial length, the risk that the final axial length becomes excessively long may be relatively low. Therefore, hyperopia acts as a kind of buffer to lower the risk of myopia. The older the age, the lower the probability of hyperopia, hence the risk of myopia may be high. For this reason, age and SE progression risk may have been correlated in this study. Another reason may be due to emmetropization occurring around the age of 6 years. Emmetropization can be influenced by both environmental and genetic factors and might have a protective effect not only against myopia or hyperopia. If the axial length is long, emmetropia can be obtained through compensation such as lens thinning, and this process mostly occurs at a young age [29]. It is well known that refractive error stability occurs in children under the age of six, with a trend toward hyperopic regression [30]. In general, from birth to around the age of six, the proportion of hyperopia decreased and the proportion of emmetropia increased, whereas the proportion of myopia showed little difference. This is the effect of emmetropization, and it is known that the incidence of myopia increases after the age of six. According to Matsumura and Hirai’s study, when examining the refractive status of children aged 3–17 years, myopia rarely occurs until the age of six, whereas the prevalence of myopia increases from the age of seven [31]. Since the age of the patients included in this study was 4–10 years, the progression of SE was presumed to be suppressed by emmetropization in some younger children, whereas the progression of SE was greater in older children.

Due to the inconvenience and time constraints caused by CR, several studies have been conducted on whether NCR and visual acuity can be substituted for CR. Sankaridurg et al. [32] reported that when analyzing the NCR value together with uncorrected visual acuity and age, it was possible to distinguish myopia in up to 97.5% of patients. In particular, the threshold for NCR myopia was studied in a large-scale survey of myopia prevalence. Gopalakrishnan et al. reported that a threshold of SE ≤ -0.75 D agrees well for the estimation of myopia among children when using NCR instead of the traditional myopia criterion of SE ≤ -0.50 [33]. But to date, no study has examined the threshold for NCR hyperopia, that is, the upper limit of emmetropia. In this study, 0.20 D was calculated as the NCR cut-off value to distinguish between children with cycloplegic emmetropia and cycloplegic hyperopia. Therefore, under non-cycloplegic conditions, our result is meaningful as a reference value. However, as only a small sample size was included in this study, additional studies using a larger sample size are needed.

There were some limitations in the present study. First is a relatively small sample size. As a result, generalization of the conclusion is limited to a certain extent. Hence, further studies with larger sample sizes are warranted. Second, this hospital-based study tended to enroll children with higher socioeconomic status than the general population, and their parents presumably paid more attention to their child’s visual health, so it is possible that these factors acted as an unknown bias in the progression of SE. Finally, no analysis about other known risk factors for myopia occurrence and progression, such as outdoor activity duration, near work time, and parents’ refractive error status, were performed. Despite these limitations, the strengths of our study are that we analyzed emmetropia with NCR, which is different from previous studies, and also that longitudinal SE changes were analyzed over a 2-year follow-up.

## Conclusion

Even if NCR showed emmetropia, patients with emmetropia with CR, showed more SE progression over 2 years than those with hyperopia with CR. SE progression was greater with age, baseline CR values indicating less hyperopia, and smaller baseline CR-NCR differences. Therefore, even if NCR shows emmetropia, the prognosis of SE progression may differ depending on the CR value, and CR must be performed when diagnosing refractive errors and predicting SE progression in children.

## Data Availability

The datasets generated during and analyzed during the current study are not publicly available due to institutional database access restrictions but are available from the corresponding author on reasonable request.
